# Assessing Neonatal Pain with NIPS and COMFORT-B: Evaluation of NICU's Staff Competences

**DOI:** 10.1155/2022/8545372

**Published:** 2022-03-16

**Authors:** Eliza Sarkaria, Dariusz Gruszfeld

**Affiliations:** Department of Neonatology, Children's Memorial Health Institute, Warsaw, Poland

## Abstract

**Background:**

Pain is considered “the 5th vital sign” that should be regularly assessed in the neonatal intensive care setting. Although over 40 pain assessment tools have been developed for neonates, their implementation in everyday practice is challenging. Epidemiological studies demonstrate that pain is still underassessed and undertreated in European NICUs.

**Purpose:**

To evaluate the interrater and intrarater reliability of the NIPS and COMFORT-B scales among the tertiary NICU's staff members 4 years after their implementation in local pain guidelines with no prior dedicated training.

**Methods:**

Physicians and nurses were invited to evaluate 5 video recordings of infants hospitalized in the intensive care settings, using the NIPS and COMFORT-B scales. The assessment took part twice at a 3-month interval. Interrater reliability was calculated for both scales using Kendall's W coefficient of concordance and Krippendorff's alpha coefficient. Cohen's kappa was used to assess intrarater reliability.

**Results:**

17 physicians and 19 nurses took part in the study. Interrater agreement for the COMFORT-B scale was above 0.8 for Kendall's W coefficient (*p* < .01) and above 0.667 for Krippendorff's alpha coefficient. Kendall's W coefficient for the NIPS scores ranged between 0.7 and 0.8 (*p* < .01). Krippendorff's alpha was above 0.667. Intrarater agreement for both the COMFORT-B and NIPS scales was 0.693 and 0.724, respectively.

**Conclusions:**

Overall, the agreement between our staff members was moderately good for both scales. This is not enough to avoid inadequate pain assessment. More training is needed to improve NICU's staff competences in using pain scales.

## 1. Introduction

Over 30 years ago, a study published by Anand et al. [1] demonstrated that inadequate analgesia during surgery in preterm babies resulted in more pronounced metabolic stress response and unstable clinical course in the postoperative period. Hence, the myth that the immature central nervous system precludes neonates from experiencing pain was rejected. Since then, neonatal pain research has made a considerable progress in understanding the developmental aspects of postnatal nociception [2]. In the 1990s, distinct behavioural and physiological responses to painful stimuli were characterized by Craig et al. [3], which led to the development of numerous neonatal pain assessment tools. To date, over 40 scales to assess pain and/or sedation in neonates have been created, yet there is still no gold standard instrument [4]. Clinical guidelines on neonatal pain prevention and management [5–7] recommend to use pain scales with proven validity and reliability such as Neonatal Facial Coding System (NFCS), Premature Infant Pain Profile (PIPP), Neonatal Pain, Agitation and Sedation Scale (N-PASS), Behavioural Infant Pain Profile (BIIP), Douleur Aiguë du Nouveau-né (DAN), COMFORT scale, and Face, Legs, Activity, Cry, Consolability (FLACC) scale. The assessment method should be adapted to the type of pain a neonate is experiencing, namely, acute, prolonged, or postoperative pain. Pain should be evaluated and documented every 4 to 6 hours and after each potentially painful procedure [7].

It has been demonstrated that implementing guidelines in everyday practice is challenging. In the EUROPAIN (EUROpean Pain Audit In Neonates) prospective observational study performed in 243 NICUs from 18 European countries [8], 31.8% of enrolled neonates received an assessment of continuous pain at least once during their NICU stay. Daily pain assessments occurred in only 10.4% of patients. It is notable that practices varied among countries with the common occurrence of pain assessment in French (100%), Dutch (80%), and Belgian (75%) NICUs. As for Polish NICUs, 2 (25%) out of 8 hospitals participating in the study reported performing continuous pain assessment. It was demonstrated that the presence of local NICU pain guidelines and nurses that specialized in pain management increased the odds for pain assessment.

In the Children's Memorial Health Institute in Warsaw, whose NICU also participated in the EUROPAIN study, there is a local guideline document regarding pain management. Medical charts are occasionally audited by the pain management services to verify that pain assessment is performed. Their staff also provides support in pharmacotherapy, if needed. In our standard of care, neonatal pain assessment is performed with the use of the Neonatal Infant Pain Scale (NIPS) in nonventilated patients and the COMFORT Behaviour (COMFORT-B) scale in ventilated patients. Nurses are provided with cards describing each scale at their workstations.

Unlike many reports on the implementation of pain assessment in hospital settings [9, 10], pain scales were introduced in our department without prior extensive training or calculation of interrater reliability. To our knowledge, their Polish translations did not undergo cross-cultural adaptation and validation. Yet, since their introduction in 2017, they have been meticulously documented in medical records. To improve our pain awareness and pain measurement, we conducted a study with the aim to evaluate the agreement between observers using both scales.

## 2. Materials and Methods

This was a prospective study conducted from January to April 2021 in the level 3 NICU of the Children's Memorial Health Institute in Warsaw. The study was approved by the local Institutional Review Board (study ID number: 21/KBE/2018).

### 2.1. Population and Design

At the time, 77 nurses and 28 doctors were employed at the NICU. They were informed about the aim of the study and their role in it during a staff meeting. Those who did not attend the meeting were personally approached by the authors. Participation in the study was voluntary. The study procedure involved the evaluation of 5 video recordings of infants hospitalized in the intensive care settings, using the NIPS and COMFORT-B scales. Participants had 2 minutes to assess each video. The assessment took part twice at a 3-month interval. At each occasion, the assessment took place after the morning staff meeting in our department's conference room.

The approximation of the minimum required sample size was based on Krippendorff's estimations [11]. We assumed that, for the NIPS, each of its 8 values (from 0 to 7) is equally likely to occur. In order to achieve the smallest acceptable reliability value of alpha 0.667 at the 0.05 level of statistical significance, the minimum reliability sample size was 71 units. It means that with a fixed number of 5 videos to evaluate, we had to enroll in the study at least 13 raters. Additionally, we decided to increase the minimum number of observations made in this study to at least 100 based on the recommendations of the COSMIN Checklist [12], which rates a sample of over 100 as “excellent”. It means that at least 20 raters had to be enrolled in the study. This sample should be sufficient to detect a value of Kendall W coefficient of 0.8 (*ρ*s1) with 80% power at the 0.05 level of significance, assuming that the null value (*ρ*s0) equals 0.6 [13].

### 2.2. Video Recordings

A convenience sample of 5 videos was selected to be evaluated by the study participants. Our aim was to ensure that participation in the study would not collide with staff's everyday duties, hence the small number of videos to assess.

4 of the videos were retrieved from the COMFORT Behaviour Scale instructional website (https://comfortassessment.nl/) [14]. The website provides video guides on how to evaluate each of the scale's items, as well as training videos for the full assessment. The videos selected for the study included: video 1: COMFORT score of 19/20; video 2: extreme scores (5) for “Calmness,” “Alertness,” “Respiratory response,” and “Physical movement”; video 3: score 5 for “Crying”; video 4: score 3 for “Respiratory response”.

The 5^th^ video was recorded in our department presenting a full-term neonate undergoing a venepuncture procedure, which is classified as moderately painful [15]. Written parental consent was obtained before the recording, and the venepuncture was clinically necessary.

### 2.3. Pain Assessment

The Neonatal Infant Pain Scale (NIPS) is a tool developed in the early 1990s [16] aimed to assess six behavioural reactions to painful procedures in preterm and full-term newborns. The scale was demonstrated to have high interrater reliability and internal consistency. It was validated for construct and concurrent validity. Its recommended use is for acute and postoperative pain, although its psychometric studies were mainly validated for acute pain [5]. It contains six items defined in Table 1. In order to provide the total NIPS score, participants in the study had to evaluate all of the items.

The COMFORT scale was developed to assess the levels of distress in PICU patients, as well as postoperative pain in children under 3 years of age. It consists of six behavioural items and two physiologic items: heart rate and mean arterial pressure. As physiological variables were demonstrated to have a weak correlation with pain behaviour, their exclusion from the scale led to creating the COMFORT-B scale containing only behavioural items. The scale is illustrated in Table 2. It is possible to omit one of the scale's items in the pain assessment. The total score is then computed by multiplying the total score for the other items by 6/5 [14]. The scale was validated for concurrent validity, internal consistency, and interrater reliability [17, 19, 20].

### 2.4. Statistical Analyses

The data were analysed using IBM SPSS Statistics v. 27. Descriptive statistics were used to calculate median scores and interquartile ranges. Kendall's W and Krippendorff's alpha coefficients were calculated to evaluate interrater reliability (IRR) for COMFORT-B and NIPS total scores, as well as for items of each scale. Both coefficients are suitable for ordinal ratings with more than 2 raters [21]. For interpretation of coefficients, we assumed the labels suggested by Landis and Koch for the use of kappa: values between 0 and 0.20 indicate a slight IRR; values between 0.21 and 0.40 indicate a fair IRR; values between 0.41 and 0.60 indicate a moderate IRR; values between 0.61 and 0.80 indicate a substantial IRR; and values between 0.81 and 1.00 indicate an almost perfect IRR [22]. Additionally, for Krippendorff's alpha, it is accepted that its lowest conceivable limit is 0.667 [11]. Intrarater reliability was assessed using Cohen's kappa coefficient. Where applicable, tests were performed at 0.05 significance level. Missing values were omitted from the analyses.

## 3. Results

36 members of our NICU staff took part in our study. The group included 5 doctors and 9 nurses with less than 5 years' experience in a neonatal intensive care unit. The remaining 12 doctors and 10 nurses had more than 5 years' of NICU experience.

**Figure 1:**
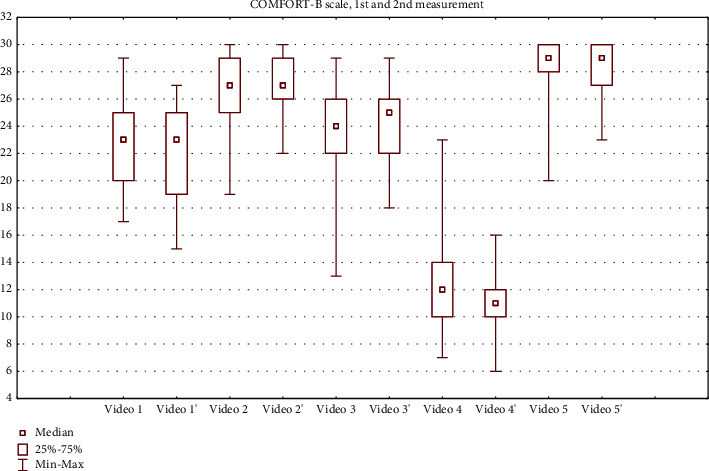
COMFORT-B scale scores. 2^nd^ measurements are marked with'.

**Figure 2:**
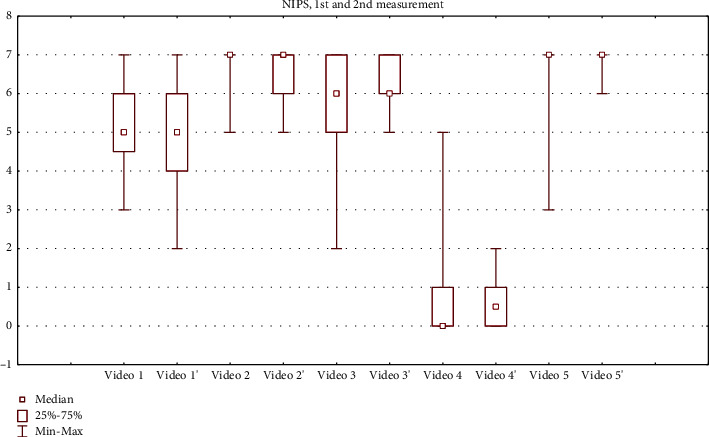
NIPS scores. 2^nd^ measurements are marked with'.

We obtained 180 and 170 total NIPS scores at the 1^st^ and 2^nd^ measurements, respectively. Total COMFORT-B scores amounted to 175 at both measurements. Total scores for all assessments are displayed as box and whisker plots (Figures 1 and 2). The percentage of observers who assessed 4 videos exactly as in reference from the COMFORT training website is illustrated in Table 3. As for the 5^th^ video that showed a procedure considered to be moderately painful, the total scores displayed in Figures 1 and 2 are within a range of severe pain.

### 3.1. Interrater Reliability: Total Scores

Interobserver agreement for the COMFORT-B and NIPS scales is presented in Tables 4 and 5, respectively. Kendall's W coefficients values (from 0.736 to 0.906) indicate substantial to almost perfect agreement between observers. Krippendorff's alpha coefficients are above the smallest acceptable value of 0.667, but below 0.8, which implies moderate interrater reliability. All reliability coefficients achieved higher values for the COMFORT-B scale and for the 2^nd^ measurement in both scales.

### 3.2. Interrater Reliability: Scales' Items

Interobserver agreement for the COMFORT-B and NIPS scales's items is presented in Tables 6 and 7. Overall, the values of reliability coefficients seem to be more consistent for the NIPS scores. The items that did not reach the minimum desired level of interrater reliability include “Breathing pattern” (both coefficients) and “Legs”(alpha). The observers showed almost perfect agreement (Kendall's W and Krippendorff's alpha >0.8) while assessing the following items of the NIPS: “Facial expression,” “Cry,” and “State of Arousal”.

As for the COMFORT-B scale, Kendall's W coefficients were shown to be above the substantial agreement threshold for all items, but they rarely reached a value greater than 0.8. However, Krippendorff's alpha coefficients were below the acceptable agreement level for the following items of the COMFORT-B scale: “Alertness,” “Respiratory response,” “Crying,” “Muscle tone,” and “Facial tension”.

### 3.3. Intrarater Reliability: Total Scores

Intrarater reliability calculated as Cohen's kappa weighted coefficients is of substantial value for both COMFORT-B and NIPS: 0.693 (CI: 0.637–0.750, *p* < .01) and 0.724 (CI: 0.658–0.791, *p* < .01), respectively.

## 4. Discussion

In this study, we evaluated agreement between a sample of our staff members in pain assessment using the NIPS and COMFORT-B scales 4 years after their introduction in our department. Contrary to other studies [9, 10, 14, 23, 24], we did not undergo intensive training before implementing these tools into our everyday practice. We only received cards describing each scale that are available at nurses' workstations. Our lack of training could explain why such a low percentage of our group assessed the videos in accordance with the COMFORT training website. Nevertheless, in our group, we showed to have moderately good inter- and intrarater agreements for both scales, which indicates that, within our department, we can communicate with each other about patients' pain levels. Calculated values of reliability coefficients were slightly lower for nurses than for doctors. That could be explained by the higher prevalence of professionals with more work experience in neonatal intensive care in the doctors' group.

The results of the scales' items analysis are more conflicting. If we take into consideration only Krippendorff's alpha coefficients, we failed to demonstrate interrater agreement in 5 out of 7 items of the COMFORT scale and in 2 out of 5 items of the NIPS. It can be speculated that these results are due to our lack of training and also technical difficulties related to applying some of these items to a video recording (e.g., evaluation of muscle tone or respiratory response). It is worth noting that results were more consistent for the NIPS scores where we collected the same number of observations for all items compared to the COMFORT-B where items differed in the number of observations. It is likely that our sample size in the COMFORT-B scale's item analysis was inadequate for the estimation of Krippendorff's alpha coefficients [25].

We used two different reliability coefficients that are suitable for ordinal ratings with more than 2 raters. They are based on different mathematical assumptions, which leads to providing different numerical values for the same datasets [26]. Krippendorff's alpha is considered to be a conservative measure of reliability favouring more even distribution inferred as the pattern by which cases fall into categories [26]. Kendall's W coefficient measures the associations between ratings with no assumptions regarding the nature of the probability distribution [27]. It is worth noting that all of Kendall's W statistics reached a significance of *p* < .01. Moreover, for the interpretation of Kendall's W coefficient, we employed Landis and Koch benchmarks [22] that were originally designed for Cohen's kappa and are the most widely used in research. However, it is not certain whether they should be applied with regard to coefficients based on different assumptions than kappa [28]. In the studies related to pain assessment scales in neonates Cohen's kappa, linearly weighted Cohen's kappa and intraclass coefficient were the most widely used [4]. We are convinced that the reliability measures we chose to apply in our study are suitable for the dataset we had to analyse [28]. However, we are aware that selecting them instead of kappa statistics precludes from comparisons of our results with other studies involving pain assessment in neonates [12].

To our knowledge, there has been only one study comparing the NIPS and COMFORT-B scales [29]. It demonstrated that while evaluating painful procedures, the NIPS has a significantly higher coefficient of variation (CV, 188% ± 99%) compared to the COMFORT scale (33% ± 8%). We did not identify any studies comparing the interrater reliability of both scales. However, they were used together as endpoints in several randomised controlled trials [30–33].

The main limitation of our study is that the relative representation of nurses in our group is much smaller compared to doctors. Only 19 of 77 employed at that time nurses took part in our study, whereas the group of doctors included 17 of 28 employed physicians. In our department, pain assessment is part of nurses' responsibilities. In case of elevated pain scores, the adjustments of pharmacological treatment are discussed with doctors. Therefore, it is essential for physicians to be familiar with the pain scales used in NICU. In our study, Kendall's W coefficients indicated almost perfect interrater agreement among doctors and substantial agreement among nurses. Given the importance of pain assessment, it should be our aim to achieve agreement above 0.8 between nurses. The results of our study imply there is a need for more training in using pain assessment tools.

The strength of our study is that it shows the real-life experience of a tertiary NICU, where the strain of everyday duties and work overload leads at times to omission of training in matters that seem to be intuitive and less vital than life-saving procedures. There is growing evidence that early life exposure to painful stimuli leads to long-term consequences such as altered pain sensitivity [34–37], impaired cognitive, behavioural, and motor development [38], and structural changes in the central nervous system detected in MRI studies [39–41]. As much as there is no doubt that pain prevention and management are crucial in neonatal care, the introduction of pain assessment tools in everyday practice is a challenge. Newborns hospitalized in NICUs are affected by different types of pain, namely, acute, postoperative, and prolonged pain. Most of the available pain scales were validated for acute pain, while tools for the evaluation of prolonged pain are scarce. Moreover, it is known that the severity of illness may affect the pain expression in neonates. Given that most behavioural pain scales are based on pain expression indices, it has not been established yet whether the cutoff values used for pain assessment should be different for more severely ill patients [42]. It is evident that the “one-size-fits-all” approach to pain assessment in neonates is unsatisfactory. Staff members should be trained to recognise different types of pain in a given clinical context and apply assessment tools accordingly. However, some scales require the evaluation of so many parameters that it makes it difficult for a single caregiver to measure them accurately. In other cases, the intensive care setting involving tubes and tapes covering patients' faces precludes from appropriate assessment of facial expressions. Furthermore, the main goal of pain assessment is to intervene with pain-alleviating treatment when needed. A study conducted in New York showed that pain scores documented in medical charts did not influence analgesic medication practices [43].

Some argue that there is no evidence that using standardized pain assessment tools improves patient outcomes [44]. Thus, efforts should be more focused on pain detection in everyday practice, while validated tools should be reserved for research purposes [45]. It is also advisable to engage parents in pain assessment, as they might be more motivated to detect pain than healthcare workers [46, 47]. Until better pain assessment tools are available, it is in the best interest of NICU's patients that healthcare providers focus on pain detection combined with improvement of their competence in using validated pain scales. The latter may be achieved by regular training with evaluation of interrater reliability among staff members. We believe this study to be a starting point for us to improve our pain assessment with the use of both scales.

## 5. Conclusions

Results of our study demonstrate that implementing pain scales without prior training may lead to a moderately good interrater agreement among staff members. Reliability values estimated here are not high enough to avoid inadequate pain assessment. Therefore, the development of a dedicated training programme is essential to improve our daily practice. Education should be focused on items of both scales that we identified to yield the most inconsistent scores among our staff members.

## Figures and Tables

**Table 1:** Scoring and interpretation for the NIPS [16].

Facial expression	0	Relaxed
	1	Grimace
Cry	0	No cry
	1	Whimper (mild moaning or intermittent)
	2	Vigorous crying or silent cry (based on facial movements if intubated)
Breathing pattern	0	Relaxed
	1	Change in breathing (irregular, increased, gagging, breath holding)
Arms	0	Relaxed
	1	Flexed/extended (tense straight arms, rigid and/or rapid extension)
Legs	0	Relaxed
	1	Flexed/extended (tense straight legs, rigid and/or rapid extension)
State of arousal	0	Sleeping/awake (quiet, peaceful, settled)
	1	Fussy (alert, restless, and thrashing)
NIPS score interpretation		0–1: no pain; 2: mild pain; 3–4: moderate pain; 5–7: severe pain

**Table 2:** Scoring and interpretation for the COMFORT-B scale. When performing the assessment, the infant is observed for 2 minutes. The healthcare professional must be in a position that permits a full view of the infant's face and body [17, 18].

Alertness	1	Deeply asleep (eyes closed, no response to changes in the environment)
	2	Lightly asleep (eyes mostly closed, occasional responses)
	3	Drowsy (child closes his/her eyes frequently, less responsive to the environment)
	4	Awake and alert (child responsive to the environment)
	5	Awake and hyperalert (exaggerated responses to environmental stimuli)
Calmness/agitation	1	Calm (child appears serene and tranquil)
	2	Slightly anxious (child shows slight anxiety)
	3	Anxious (child appears agitated but remains in control)
	4	Very anxious (child appears very agitated, just able to control)
	5	Panicky (severe distress with loss of control)
Respiratory response (only in mechanically ventilated children)	1	No spontaneous respiration
	2	Spontaneous and ventilator respiration
	3	Restlessness or resistance to ventilator
	4	Actively breathes against ventilator or coughs regularly
	5	Fights ventilator
Crying (only in spontaneously breathing children)	1	Quiet breathing, no crying sounds
	2	Occasional sobbing or moaning
	3	Whining (monotonous sound)
	4	Crying
	5	Screaming or shrieking
Physical movement	1	No movement
	2	Occasional, (three or fewer) slight movements
	3	Frequent, (more than three) slight movements
	4	Vigorous movements limited to extremities
	5	Vigorous movements including torso and head
Muscle tone	1	Muscles totally relaxed; no muscle tone
	2	Reduced muscle tone; less resistance than normal
	3	Normal muscle tone
	4	Increased muscle tone and flexion of fingers and toes
	5	Extreme muscle rigidity and flexion of fingers and toes
Facial tension	1	Facial muscles totally relaxed
	2	Normal facial tone
	3	Tension evident in some facial muscles (not sustained
	4	Tension evident throughout facial muscles (sustained)
	5	Facial muscles contorted and grimacing
COMFORT-B score interpretation		Sedation levels: <10 oversedation, >23 undersedation [17]
		Pain >17 along with the numeric rating scale (NRS) > 4 indicate pain [18] NRS can be substituted for any validated pain tool

**Table 3:** COMFORT score in relation to reference videos (training website: https://comfortassessment.nl/, [14]).

	Reference score	*N* at 1^st^ assessment	*N* at 2^nd^ assessment
Video 1	19/20	6 (17%)	9 (25%)
Video 2	5 for calmness	12 (35%)	14 (40%)
	5 for alertness	31 (88%)	31 (88%)
	5 for respiratory response	14 (40%)	15 (42%)
	5 for physical movement	26 (74%)	29 (82%)
Video 3	5 for crying	10 (28%)	4 (11%)
Video 4	3 for respiratory response	4 (11%)	1 (3%)

**Table 4:** COMFORT-B scale total score: interrater reliability.

	Kendall's W coefficient *p* < .01		Krippendorff's alpha coefficient (95% confidence interval)	
	1^st^ measurement	2^nd^ measurement	1^st^ measurement	2^nd^ measurement
All observers *N* = 35	0.807	0.877	0.6899 [0.6719–0.7081]	0.7414 [0.7279–0.7554]
Doctors *N* = 17	0.851	0.906	0.7231 [0.6870–0.7559]	0.7846 [0.7622–0.8084]
Nurses *N* = 18	0.783	0.853	0.6896 [0.6514–0.7271]	0.6866 [0.6557–0.7189]

*N*: number of observers.

**Table 5:** NIPS total score: interrater reliability.

	Kendall's W coefficient < 0.01		Krippendorff's alpha coefficient (95% confidence interval)	
	1st measurement	2^nd^ measurement	1^st^ measurement	2^nd^ measurement
All observers	0.766 (*N* = 36)	0.807 (*N* = 34)	0.6737 [0.6552–0.6927]	0.7302 [0.7151–0.7467]
Doctors	0.818 (*N* = 17)	0.869 (*N* = 16)	0.7488 [0.7138–0.7816]	0.7640 [0.7310–0.7951]
Nurses	0.736 (*N* = 19)	0.786 (*N* = 18)	0.6231 [0.5844–0.6606]	0.7244 [0.6971–0.7612]

*N*: number of observers.

**Table 6:** COMFORT-B scale, interrater agreement item analysis.

	Kendall's W coefficient *p* < .01		Krippendorff's alpha coefficient (95% confidence interval)	
	1^st^ measurement	2^nd^ measurement	1^st^ measurement	2^nd^ measurement
Alertness (*N* = 34**)**	0.632	0.513	0.4979 [CI: 0.4702–0.5252]	0.3987 [CI: 0.3676–0.4302]
Calmness/agitation (*N* = 33**)**	0.773	0.872	0.7003 [CI: 0.6827–0.7178]	0.7776 [CI: 0.7652–0.7891]
Respiratory response (only in mechanically ventilated children) (*N* = 35**)**	0.690	0.886	0.3888 [CI: 0.3407–0.4327]	0.5919 [CI:0.5569–0.6247]
Crying (only in spontaneously breathing children) (*N* = 34**)**	0.720	0.776	0.5213 [CI: 0.4864–0.5573]	0.6685 [CI: 0.6381–0.6948]
Physical movement (*N* = 31)	0.781	0.805	0.6720 [CI: 0.6512–0.6938]	0.6917 [CI: 0.6696–0.7128]
Muscle tone (*N* = 34)	0.617	0.722	0.5048 [CI 0.4780–0.5315]	0.5963 [CI: 0.5744–0.6201]
Facial tension (*N* = 34)	0.700	0.755	0.5490 [CI: 0.5250–0.5716]	0.5946 [CI: 0.5713–0.6163]

*N*: number of observers.

**Table 7:** NIPS, interrater agreement item analysis (*N* = 34 in all cases).

	Kendall's W coefficient *p* < .01		Krippendorff's alpha (95% confidence interval)	
	1^st^ measurement	2^nd^ measurement	1^st^ measurement	2^nd^ measurement
Facial expression	0.863	0.884	0.8304 [CI: 0.8044–0.8553]	0.8539 [CI: 0.8272–0.8782]
Cry	0.809	0.824	0.7359 [CI: 0.7157–0.7553]	0.7758 [CI: 0.7571–0.7942]
Breathing pattern	0.312	0.372	0.2451 [CI: 0.2074–0.2821]	0.2826 [CI: 0.2447–0.3220]
Arms	0.658	0.772	0.5780 [CI: 0.5430–0.6121]	0.7140 [CI: 0.6840–0.7430]
Legs	0.677	0.738	0.5587 [CI 0.5241–0.5933]	0.6519 [CI: 0.6208–0.6839]
State of arousal	0.854	0.856	0.8060 [CI: 0.7784–0.8315]	0.8199 CI: 0.7927–0.8459]

## Data Availability

The data used to support the findings of this study are available from the corresponding author (e.sarkaria@ipczd.pl) upon request.

## References

[1] Anand K. J. S., Sippell W. G., Aynsley-Green A. (1987). Randomised trial of fentanyl anaesthesia in preterm babies undergoing surgery: effects on the stress response. *The Lancet*.

[2] Fitzgerald M. (2015). What do we really know about newborn infant pain?. *Experimental Physiology*.

[3] Craig K. D., Whitfield M. F., Grunau R. V. E., Linton J., Hadjistavropoulos H. D. (1993). Pain in the preterm neonate: behavioural and physiological indices. *Pain*.

[4] Giordano V., Edobor J., Deindl P. (2019). Pain and sedation scales for neonatal and pediatric patients in a preverbal stage of development: a systematic review. *JAMA Pediatrics*.

[5] Statement P. (2016). Prevention and management of procedural pain in the neonate: an update. *Pediatrics*.

[6] Harris J., Ramelet A.-S., van Dijk M. (2016). Clinical recommendations for pain, sedation, withdrawal and delirium assessment in critically ill infants and children: an ESPNIC position statement for healthcare professionals. *Intensive Care Medicine*.

[7] Anand K. J. S. (2001). Consensus statement for the prevention and management of pain in the newborn. *Archives of Pediatrics and Adolescent Medicine*.

[8] Anand K. J. S., Eriksson M., Boyle E. M. (2017). Assessment of continuous pain in newborns admitted to NICUs in 18 European countries. *Acta Paediatrica*.

[9] Gallo A.-M. (2003). The fifth vital sign: implementation of the Neonatal Infant Pain Scale. *Journal of Obstetric, Gynecologic, and Neonatal Nursing*.

[10] Stenkjaer R. L., Pedersen P. U., Hundrup Y. A., Weis J. (2019). Evaluation of NICU nurses’ competence in pain assessment 5 Years after implementation of the COMFORTneo scale. *Advances in Neonatal Care*.

[11] Krippendorff K. (2004). *Content Analysis: An Introduction to its Methodology*.

[12] Mokkink L. B., Terwee C. B., Patrick D. L. (2010). The COSMIN checklist for assessing the methodological quality of studies on measurement properties of health status measurement instruments: an international Delphi study. *Quality of Life Research*.

[13] May J. O., Looney S. W. (2020). Sample size charts for Spearman and Kendall coefficients. *Journal of Biometrics & Biostatistics*.

[14] Van Dijk M., Peters J. W. B., Van Deventer P., Tibboel D. (2005). The COMFORT behavior scale: a tool for assessing pain and sedation in infants. *AJN, American Journal of Nursing*.

[15] Laudiano-Dray M. P., Pillai Riddell R., Jones L. (2020). Quantification of neonatal procedural pain severity: a platform for estimating total pain burden in individual infants. *Pain*.

[16] Lawrence J., Alcock D., Kay J., McGrath P. J. (1991). The development of a tool to assess neonatal pain. *Journal of Pain and Symptom Management*.

[17] Ista E., Van Dijk M., Tibboel D., De Hoog M. (2005). Assessment of sedation levels in pediatric intensive care patients can be improved by using the COMFORT behavior scale. *Pediatric Critical Care Medicine*.

[18] Boerlage A. A., Ista E., Duivenvoorden H. J., De Wildt S. N., Tibboel D., Van Dijk M. (2015). The COMFORT behaviour scale detects clinically meaningful effects of analgesic and sedative treatment. *European Journal of Pain*.

[19] Van Dijk M., De Boer J. B., Koot H. M., Tibboel D., Passchier J., Duivenvoorden H. J. (2000). The reliability and validity of the COMFORT scale as a postoperative pain instrument in 0 to 3-year-old infants. *Pain*.

[20] Ambuel B., Hamlett K. W., Marx C. M., Blumer J. L. (1992). Assessing distress in pediatric intensive care environments: the comfort scale. *Journal of Pediatric Psychology*.

[21] ten Hove D., Jorgensen T. D., van der Ark L. A., Wiberg M., Culpepper S., Janssen R., González J., Molenaar D. (2018). *On the Usefulness of Interrater Reliability Coefficients BT - Quantitative Psychology*.

[22] Landis J. R., Koch G. G. (1977). The measurement of observer agreement for categorical data. *Biometrics*.

[23] Dunbar A. E., Sharek P. J., Mickas N. A. (2006). Implementation and case-study results of potentially better practices to improve pain management of neonates. *Pediatrics*.

[24] Desai A., Aucott S., Frank K., Silbert-Flagg J. (2018). Comparing N-PASS and NIPS: improving pain measurement in the neonate. *Advances in Neonatal Care*.

[25] Krippendorff K. (2011). Agreement and information in the reliability of coding. *Communication Methods and Measures*.

[26] Zhao X., Liu J. S., Deng K. (2013). Assumptions behind intercoder reliability indices. *Annals of the International Communication Association*.

[27] John N. L., Maurice M. A., Kendall G. (1949). *Rank Correlation Methods*.

[28] Ark V. D. (2018). *UvA-DARE ( Digital Academic Repository ) on the Usefulness of Interrater Reliability Coefficients on the Usefulness of Interrater Reliability Coefficients*.

[29] Blauer T., Gerstmann D. (1998). A simultaneous comparison of three neonatal pain scales during common NICU procedures. *The Clinical Journal of Pain*.

[30] Silva Y. P. e., Gomez R. S., de Oliveira Marcatto J., Maximo T. A., Barbosa R. F., Silva A. C. S. e. (2007). Morphine versus remifentanil for intubating preterm neonates. *Archives of Disease in Childhood - Fetal and Neonatal Edition*.

[31] Kahraman A., Başbakkal Z., Yalaz M., Sözmen E. Y. (2018). The effect of nesting positions on pain, stress and comfort during heel lance in premature infants. *Pediatrics & Neonatology*.

[32] Penido M. G., de Oliveira Silva D. F., Tavares E. C., e Silva Y. P. (2011). Propofol versus midazolam for intubating preterm neonates: a randomized controlled trial. *Journal of Perinatology*.

[33] Silva E., Gomez R. S., Marcatto J. D. E. O., Maximo T. A., Barbosa R. F. (2008). Early awakening and extubation with remifentanil in ventilated premature neonates. *Pediatria Anesthecia*.

[34] Taddio A., Katz J., Ilersich A. L., Koren G. (1997). Effect of neonatal circumcision on pain response during subsequent routine vaccination. *The Lancet*.

[35] Peters J. W. B., Schouw R., Anand K. J. S., van Dijk M., Duivenvoorden H. J., Tibboel D. (2005). Does neonatal surgery lead to increased pain sensitivity in later childhood?. *Pain*.

[36] Valeri B. O., Ranger M., Chau C. M. Y. (2016). Neonatal invasive procedures predict pain intensity at school age in children born very preterm. *The Clinical Journal of Pain*.

[37] Walker S. M., Melbourne A., O’Reilly H. (2018). Somatosensory function and pain in extremely preterm young adults from the UK EPICure cohort: sex-dependent differences and impact of neonatal surgery. *British Journal of Anaesthesia*.

[38] Vinall J., Miller S. P., Bjornson B. H. (2014). Invasive procedures in preterm children: brain and cognitive development at school age. *Pediatrics*.

[39] Ranger M., Celeste Johnston C., Rennick J. E., Limperopoulos C., Heldt T., Du Plessis A. J. (2013). A multidimensional approach to pain assessment in critically ill infants during a painful procedure. *The Clinical Journal of Pain*.

[40] Duerden E. G., Grunau R. E., Guo T. (2018). Early procedural pain is associated with regionally-specific alterations in thalamic development in preterm neonates. *Journal of Neuroscience*.

[41] Smith G. C., Gutovich J., Smyser C. (2011). Neonatal intensive care unit stress is associated with brain development in preterm infants. *Annals of Neurology*.

[42] Van Dijk M., Tibboel D. (2012). Update on pain assessment in sick neonates and infants. *Pediatric Clinics of North America*.

[43] Rohan A. J. (2014). The utility of pain scores obtained during ’regular reassessment process’ in premature infants in the NICU. *Journal of Perinatology*.

[44] Franck L. S., Bruce E. (2009). Putting pain assessment into practice: why is it so painful?. *Pain Research and Management*.

[45] Bellieni C. V., Tei M., Buonocore G. (2015). Should we assess pain in newborn infants using a scoring system or just a detection method?. *Acta Paediatrica*.

[46] Xavier Balda R. d. C., Guinsburg R., Almeida M. F. B. d., Peres C. d. A., Miyoshi M. H., Kopelman B. I. (2000). The recognition of facial expression of pain in full-term newborns by parents and health professionals. *Archives of Pediatrics and Adolescent Medicine*.

[47] Pillai Riddell R. R., Craig K. D. (2007). Judgments of infant pain: the impact of caregiver identity and infant age. *Journal of Pediatric Psychology*.

